# Supplementary material for: Mismatch repair deficiency endows tumors with a unique mutation signature and sensitivity to DNA double-strand breaks
Source: eLife. 2014 Aug 1;3:e02725. doi: 10.7554/eLife.02725 (PMC4141275; doi:10.7554/eLife.02725)
Supplement: Supplementary file 1. — DOI: http://dx.doi.org/10.7554/eLife.02725.039 [file elife02725s010.pdf]

Note S1. Evidence for negative clonal selection in MMR-deficient exomes

Mutation frequencies in MMR-deficient whole-genomes *versus* exomes: Overall mutation frequencies were defined as the number of somatic mutations per base (mpb) in a given genomic region (e.g., one somatic mutation in a region of 10^6 bases corresponds to a mutation frequency of 1E-06 mpb). Overall, in the MMR-deficient whole-genome, we observed a genome-wide mutation frequency of 9.4E-05 mpb. To assess negative selection in the exome, we checked whether *i)* there was a lower mutation frequency in the exome relative to the whole-genome, and whether *ii)* the frequency of somatic mutations was more prominently decreased in the exome. We observed that the mutation frequency in the exome was indeed 26% lower than in intergenic or intronic regions. Stratification of mutation frequencies into substitutions and indels demonstrated that the substitution frequency in the exome decreased by 1.1%, whereas the decrease for indels was 91% compared to the whole-genome. As homopolymers in exomes have characteristics that differ from those in the rest of the genome in terms of number, base composition and length, we corrected indel frequencies for these confounding factors.

Correction of indel frequencies for homopolymer content, composition and length: We calculated the frequency of affected homopolymers for each genomic location (**t**: exonic, 5'UTR, 3'UTR, intronic, intergenic or genomic), for each type of homopolymer (**AT** or CG composition) and each homopolymer length (6, 7, 8, etc. (**l**).

$$^{AT}Freq_l^t = \frac{^{AT}_{aff}n_l^t}{^{AT}n_l^t}$$

Next, we calculate the relative increase of observed frequencies relative to the frequency observed at the genome-wide level:

$$^{AT}rFreq_l^t = \frac{^{AT}Freq_l^t}{^{AT}Freq_l^{genomic}}$$

The frequency $^{AT}rFreq^t_l$ was normalized for the number of homopolymers of a given length l, for each genomic location t and for homopolymer composition ($^{AT}wrFreq^t_l$), and further normalized for the number of AT (or GC) homopolymers for each genomic location and homopolymer length ($^{AT}nwrFreq^t_l$).

$$^{AT}wrFreq_l^t = {}^{AT}rFreq_l^t \times \frac{^{AT}n_l^t}{\sum_{i=6} {}^{AT}n_i^t}$$

$$^{AT}nwrFreq_l^t = {}^{AT}wrFreq_l^t \times \frac{^{AT}n_l^t}{^{AT}n_l^t + {}^{CG}n_l^t}$$

All the weighted frequencies are then summed for every genomic location ($cFreq^t$) and divided by the overall summed genomic frequency (rFreq).

$$cFreq^t = \sum_{i=6} {}^{AT}nwrFreq_i^t + \sum_{i=6} {}^{CG}nwrFreq_i^t$$

$$rFreq = \frac{cFreq^t}{cFreq^{genomic}}$$

Despite these extensive corrections, a 16% decrease in indel frequency in exonic regions persisted.

Note S2. Materials

Mouse anti-phospho-Histone H2A.X (Ser139) monoclonal antibody (clone JBW301) was from Millipore Corporation, Billerica, MA, USA. Rabbit anti-Rad51 (PC130) polyclonal antibody was from Calbiochem/Merck, Darmstadt, Germany. Rabbit anti-ACTB (#4967) polyclonal antibody was from Cell Signalling, Danvers, MA, USA. FITC-conjugated anti-BrdU antibody (347583) was from Becton-Dickinson, San Jose, CA, USA. Alexa Fluor 647 goat anti-mouse IgG (A-21235) and Alexa Fluor 488 goat anti-rabbit IgG (A-11034) were from Life technologies, Carlsbad, CA, USA. Olaparib (AZD-2281, batch JSAR104) was purchased from JS Research Chemicals Trading, Schleswig Holstein, Germany. Cis-platinum(II)diammine dichloride (P4394), paclitaxel (T7402), mitomycin C (M4287), (S)-(+)-camptothecin (C9911) and carmustine (C0400) were purchased from Sigma-Aldrich, St. Louis, MO, USA, and prepared and stored according to the manufacturer's recommendations. siRNA ON-TARGETplus SMART pools were purchased from Thermo Scientific Dharmacon, Chicago, IL, USA: Non-targeting (D-001810-10-05); ATM (L-003201-00-0005); ATR (L-003202-00-0005); BRCA1 (L-003461-00-0005); and BRCA2 (L-003462-00-0005). TaqMan gene expression assays (Life technologies, Carlsbad, CA, USA) used in this study were as follows ATM: Hs01112355_g1; ATR: Hs00992123_m1; BRCA1: Hs01556193_m1; BRCA2: Hs00609073_m1; ACTB: Hs99999903_m1. Normal goat serum (005-000-121) was from Jackson Immunoresearch Labs, West Grove, PA USA.
